# Green-banana biomass consumption by diabetic patients improves plasma low-density lipoprotein particle functionality

**DOI:** 10.1038/s41598-020-69288-1

**Published:** 2020-07-23

**Authors:** Zahra Lotfollahi, Ana Paula de Queiroz Mello, Edna S. Costa, Cristiano L. P. Oliveira, Nagila R. T. Damasceno, Maria Cristina Izar, Antonio Martins Figueiredo Neto

**Affiliations:** 1 Complex Fluids Group, Instituto de Física, Universidade de São Paulo, Rua Do Matão, 1371, Butantã, São Paulo, SP CEP: 05508-090 Brazil; 2 University Center São Camilo, São Paulo, Brazil; 3 Federal University of São Paulo, São Paulo, Brazil; 4 Faculty of Public Health, University of São Paulo (USP), São Paulo, Brazil

**Keywords:** Biophysics, Cardiology, Risk factors, Physics

## Abstract

The aim of this study was to investigate the effects of 6-months consumption of green-banana biomass on the LDL particle functionality in subjects with type 2 diabetes. Subjects (n = 39, mean age 65 years old) of both sexes with diabetes (HbA1c ≥ 6·5%) were randomized to receive nutritional support plus green-banana biomass (40 g) (n = 21) or diet alone (n = 18) for 6-months. Non-linear optical responses of LDL solutions from these participants were studied by Z-scan technique. UV–visible spectrophotometer was used to measure the absorbance of the LDL samples. Small Angle X-ray Scattering and Dynamic Light Scattering experiments were used to look for any structural changes in LDL samples and to determine their size distribution. The Lipoprint test was used to determine the LDL sub-fractions in terms of distribution and size. Consumption of green-banana biomass, reduced total- (*p* = 0.010), non-HDL-cholesterol (*p* = 0.043), glucose (*p* = 0.028) and HbA1c (*p* = 0.0007), and also improved the protection of the LDL particle against oxidation, by the increase in carotenoids content in the particles (*p* = 0.007). This higher protection against modifications may decrease the risk of developing cardiovascular disease. These benefits of the green-banana biomass encourage the use of resistant starches with potential clinical applications in individuals with pre-diabetes and diabetes.

## Introduction

Type 2 diabetes mellitus (DM2) is a group of metabolic disorders characterized by hyperglycemia and is the most common form of diabetes^1^. The incidence of DM2 is increasing worldwide due to modern lifestyle, in which poor dietary habits, decreased physical activity and genetic predisposition exert synergistic effect on glucose homeostasis^2^. Epidemiological data show that about 60–90% of DM2 patients are obese. It is well known that obesity is one of the major risk factor for DM2 and cardiovascular diseases (CVD)^2–4^. With the increasing number of obese and diabetic individuals, and their impact in cardiovascular diseases, there exists an intense request for healthy diets and alimentary supplements able to improve glucose metabolism and other related dysfunctions.

Resistant starch (RS) is a form of starch that resists digestion in the small intestine and is classified as a type of dietary fiber^5^. Unripe (green) banana belongs to a RS2 class, being well known as a food with low glycemic index, non-manufactured, attractive and inexpensive food containing different kinds of fibers, vitamins, minerals and bioactive compounds with high RS content^2,3,6–8^.

In a previous work, we have shown that consumption of RS was associated with significant reduction in diastolic blood pressure, fasting glucose, glycated hemoglobin (HbA1c), body weight, body mass index (BMI), fat mass percentage, waist and hip circumferences, and increase in lean mass percentage in subjects with diabetes and pre-diabetes^9^.

On the other side, it is well established that the presence of modified Low-Density Lipoprotein (LDL) particles are one of the risk factors for developing CVD^10^. Modified LDL particles represent heterogeneous and complex sub-fractions, where physicochemical changes can disrupt functional properties of this lipoprotein with impact in atherosclerosis process.

The nonlinear optical Z-scan (ZS) technique has been shown helpful for measuring the degree of modification of LDL particles in blood, in other words, the functionality of LDL particles^11^. By functionality, we mean the characteristics of the native (i.e., non-modified, non-oxidized LDL) state of the particle. This term will be considered hereafter as the “quality” of the particle. This technique is often used in the physics of condensed matter and, more recently, was used to investigate the oxidation state of LDL particles^11,12^. The physical phenomenon present in these experiments is the formation of a thermal lens in a solution with the LDL when it is illuminated with a Gaussian laser beam. The amplitude of the thermal lens formed depends on the physicochemical state of the LDL, in which, the higher the modified (mainly oxidation) state of the particle, the smaller the amplitude of the thermal lens formed. In addition, other physical techniques such as UV–visible spectroscopy, dynamic light scattering (DLS), small angle X-ray scattering (SAXS) and the lipoprotein sub-fractions determination have been used to identify modifications of the LDL and its association with health conditions and diseases^13–15^. Recently the functionality of the LDL particles in diabetic patients with periodontal diseases was studied^16^. In patients with chronic periodontitis, after one-year of periodontal treatment, the functionality of the LDL particles was shown to be improved. This improvement was verified in the results obtained with the Z-scan technique and the linear optical absorbance in the wavelength characteristic of the carotenoids present in the particles. Despite the interest in the study of the effects of the RS2 diet^8^, to the best of our knowledge, there is not a systematic study about the benefits of the green-banana consumption in the functionality of the LDL in DM2 patients.

Therefore, the aim of this study was to address whether the consumption of RS2 from green banana biomass would affect LDL particle functionality in subjects with DM2, compared with diet alone.

Besides the usual analysis of the patients’ biochemical parameters the LDL was extracted from their plasma and analyzed with different optical and scattering experimental techniques, described in the following.

Primary endpoint of this study was the modification of LDL particle quality and functionality in patients with DM2. Secondary endpoints were the components of LDL particle that would be responsible for changes in LDL particle quality and functionality.

## Materials and methods

This study is a 6-month, prospective, randomized, open-label trial, with parallel arms and blinded endpoints. Subjects were allocated in a 1:1 ratio to each arm of the study treatment. The trial was registered on 26/07/2017 at the clinicaltrials.gov (NCT03230123), under the acronym of BIOMEL Study (Effects of Green Banana BIOmass Consumption in Patients with Pre-diabetes and Diabetes MELlitus) and can be accessed through the internet (https://clinicaltrials.gov/)^9^. The study protocol is in agreement with the Ethical Principles for Medical Research Involving Human Subjects as stated by the Declaration of Helsinki. This trial was approved by local Human Ethics Committee and all patients signed the written informed consent before data collection. For more details about the design see Costa et al.^9^.

The study was conducted at the Department of Medicine, Federal University of Sao Paulo, SP, Brazil. The recruitment process began in February 2016, and the intervention was conducted until March 2017. Briefly, patients of both sexes, aging 60–72 years, with diabetes (glycated haemoglobin ≥ 6.5%) and pre-diabetes (HbA1c between 5.7% and 6.4%, or with confirmed type 2 diabetes), receiving a stable dose of anti-hyperglycemic drugs were eligible^9^. For this subanalysis, we kept the same inclusion/exclusion criteria. We did not use the fasting glucose cut off of 126 mg/dL for inclusion because the patients were under a stable dose of anti-hyperglycemic drugs.

They were assigned to either diet intervention plus green banana biomass or diet intervention alone in a 1:1 ratio, using a random-number generator program. Thirteen patients were discontinued due to their need for insulin (six in banana group, seven in control), and five subjects in control group did not comply with the diet intervention. The final number were 21 in banana group and 18 in control group.

Patients under insulin therapy, or those that during the study needed change in dose or addition of medication for diabetes were excluded. Neoplasms, except basal-cell carcinoma, heart (NYHA class III or IV) and renal failure (e-GFR < 30 mL/min) or dialysis therapy, AIDS, uncontrolled hypothyroidism (TSH > 10 μUI/mL), active liver disease, severe psychiatric disorders, or any other disease that, in the investigator’s opinion, could interfere with the results were also excluded. Investigators who performed all analyses were blinded to interventions.

The calculation of sample size took into account variations in glycated haemoglobin (HbA1c), with a type I error alpha of 0.05 and a type II error beta of 0.2 (80% power)^9^. For this subanalysis, a convenience sample was used.

### Dietetic parameters

The diet plan was individualized according to the Brazilian Society of Diabetes, taking into account the total energy expenditure (TEE), with standardized menus for weight reduction (20–25 kcal/kg of current weight), weight maintenance (25–30 kcal/kg of current weight), and weight gain (30–35 kcal/kg of current weight), according to patient characteristics^17^. Macronutrients intake was accessed by the Acceptable Macronutrient Distribution Range^18^. Substitutions by equivalent foods were made using a food replacement list (FRL)^19^.

#### Green-banana biomass

Green-banana biomass was added to any food preparation without heating as 2 tablespoons (40 g) per day, providing ~ 4.5 g of RS. According to the manufacturer, nutrition information for green banana biomass (per 20-g portion or one tablespoon) is: carbohydrates 2.83 g, proteins 0.18 g, fiber 1.12 g, total energy expenditure 12 kcal. The resistant starch content is ~ 12%. It does not contain significant amounts of sodium and total fat, saturated, or trans fatty acids^9,20^. The product was well tolerated and did not cause discontinuation due to side effects.

#### Evaluation of food consumption

Food consumption was estimated by 24 h food records and standardized food-frequency questionnaires (FFQs) obtained at baseline (T0) and 6-months (T6), with total energy intake, macro- and micro-nutrients, lipids, cholesterol, carbohydrates, fatty acids, and vitamins calculated by the Avanutri Software (Avanutri Revolution, v. 4.0)^21–23^.

### Biochemical analysis

Fasting blood samples were obtained from all participants at T0 and T6. Commercial kits (Cobas Mira, Roche, Switzerland) were used in the analysis for blood glucose, total cholesterol (TC), glycated hemoglobin (HbA1c, %), HDL-C, and triglycerides (TG). LDL-C concentration was calculated by the Friedewald equation^24^. Ox-LDL concentration (mU/L) was determined using Mercodia Oxidized LDL—ELISA kit (Mercodia AB, Uppsala, Sweden) according to the manufacturer’s instructions. Fasting insulin was measured using the immunofluorometric assay. The HOMA-IR [fasting insulin (μUI/mL) × fasting glucose (mg/dL)/405] was calculated, with the cutoff value set at ≥ 2.8^25^.

Protein concentrations in LDL particles were determined by using the bicinchoninic acid (BCA) method, Pierce BCA Protein Assay kit (Thermo Fisher Scientific, MA, USA) with bovine serum albumin (BSA) as standard^26^.

### Low-density lipoprotein separation

Blood was collected and immediately separated in plasma and stored at − 80 °C until analysis. Low-density lipoprotein was isolated from plasma by preparative sequential ultracentrifugation (18 h, 4 °C, 105.000×*g*), using a density cut-off point of 1.063 g/mL, by ultracentrifuge equipped with a fixed-angle rotor (Hitachi Himac CP 70MX, Tokyo, Japan)^27^. These samples were dialyzed against PBS with EDTA (pH 7.4, 4 °C, 12 h, with agitation) to remove the salts.

### Z-scan technique

The Z-scan (ZS) is an experimental technique to measure nonlinear optical properties of materials^28^. In this technique, the LDL solution sample was encapsulated between two micro-slides glass with a spacer of 200 μm and is illuminated by a focused laser beam (wavelength 532 nm, power 100 mw), propagating in the z direction. A mechanical chopper provides light pulses of about 30 ms. The sample moves along the z-axis before and after the focal point z = 0 and the transmitted-laser beam is detected by a detector. The normalized transmittance as a function of the sample-z position is calculated dividing the voltage on the photodetector at each z-position of sample by the voltage when the sample is at a position far from the focal point. More details about the setup and data treatment can be found in our previous works^11,29^. The typical result in the ZS experiment is a valley to peak (or peak to valley) curve. This peak to valley amplitude ($\Delta \Gamma_{pv}$) is proportional to the phase shift (θ) of the thermal lens formed. In this study, we normalized the values of θ to compare the results from various patients because each sample had different LDL concentrations.

### UV–visible spectroscopy

The spectrophotometer measures the intensity of light passing through a sample and compares it to the intensity of the incident light beam. The linear-absorbance spectra were measured by a UV–visible spectrophotometer with light wavelength from 200 to 900 nm, using deuterium and tungsten halogen light sources and a spectrometer (USB4000, from Ocean Optics) connected to a computer for data acquisition. The samples were conditioned into a quartz cuvette, with optical path length of 1 cm. The extinction spectrum from the spectrophotometer is the sum of both the Rayleigh scattering and the absorbance. The Rayleigh scattering is proportional to λ^−4^, and its intensity is estimated for each one of the samples^29^. The absorbance is calculated by removing the scattering contribution from the extinction spectra. As is known, the LDL particle contains various molecules, and each of them has different absorption spectra^16^. For instance, the maximum light absorption of the ApoB-100 is at λ_Apo_ ≈ 280 nm^30,31^, the cholesterol at λ_chol_ < 200 nm^32^, the α-Tocopherol at λ_α-Toc_ ≈ 210 nm and the carotenoids at λ_β-Car_ ≈ λ_α-Car_ ≈440 and 480 nm^33,34^. In the present study we smeasured the absorbance values of LDL solution samples at the wavelength corresponding to the maximum of the absorbance spectrum of Carotenoids, λ = 480 nm, at baseline and after 6-months treatment.

### Small angle X-ray scattering (SAXS)

Small-angle X-ray scattering (SAXS) is a standard technique that can be used to the study of particles in solution, providing information about size, polydispersity, shape, oligomerization, flexibility and aggregate state^35,36^. SAXS data were collected in a Xenocs-XEUSS diffractometer. X-rays (wavelength λ = 1.54 Å Cu_kα_) are collimated by two sets of scatter-less slits and reaches the LDL sample placed in a cylindrical borosilicate glass capillary. This capillary is mounted on a homemade stainless-steel case, which allows an easy handling, wash and rinse. Therefore, the lipoproteins and the corresponding buffers can be measured in the same conditions. The two-dimensional scattering patterns were registered by a detector. The images were integrated with the Fit2D software and the data treatment was performed using standard procedures^35^. As a result, one obtains the scattering intensity as a function of the reciprocal space moment transfer modulus, q, defined as q = 4πsin(θ)/λ, where 2θ is the scattering angle. The data was collected at sample to detector distance of 0.90 m and the available q range is 0.015 < q < 0.45 Å^−1^.

### Dynamic light scattering (DLS)

The DLS, known as photon correlation spectroscopy, was used to assess eventual aggregation of particles and the LDL size distributions^37^. DLS measurements were carried out using a 90Plus Particle Size Analyser (Brookhaven, Holtsville, NY, USA). In this technique, the sample is illuminated by a laser beam (wavelength 657 nm and power of 35 mW) and the fluctuations of the scattered light are detected by a fast photon detector positioned at 90° from the incident light direction. The DLS measurements provide intensity correlation functions that are analyzed to determine the particle-size distribution, weighted by volume, number, and intensity of scattered light^38,39^. The fits shown in this work were obtained by using the NNLS (non-negative least squares) method^40^.

### Lipoprint system

The lipoprotein fractions (VLDL and IDL) and sub-fractions of LDL were determined by the Lipoprint system (Quantimetrix, Redondo Beach, CA), which is based on the separation and quantification of lipoprotein sub-fractions by non-denaturing polyacrylamide tube gel electrophoresis. To perform this procedure, 25 μL of the serum or plasma was added to the polyacrylamide gel tube and 200 μL of the dye-gel solution. The sample was homogenized. Then the tubes containing the samples were photo-polymerized and subjected to the electrophoresis process. After separation of the sub-fractions, the tubes were scanned in order to identify each subclass^41^. The LDL-1 and LDL-2 subclasses were classified as Large LDL and subclasses LDL-3 to LDL-7 were classified as smaller and denser particles (Small LDL). Results are shown in percentage (%) and concentration (%/total cholesterol level) of subclasses. The LDL phenotypes were based in cut-off points (phenotype A ≥ 26.8 Å and phenotype non-A < 26.8 Å)^13,14,42^. All analyses were conducted in duplicate and coefficients of variance intra and inter assay were 1–15%.

### Statistical analysis

Numerical variables were expressed as means ± Standard Deviation (SD) for normal distribution and median [Inter-Quartile Range (IQR = Q1–Q3)] for non-normal distribution. Shapiro–Wilk test was used to verify normality of the data distribution. For comparison between groups, unpaired two sample *T* test, for normal distribution or Mann–Whitney test, for non-normal distribution, were used. Within-group comparisons were carried out using the paired sample *T* test, to compare groups with normal distribution, or Wilcoxon signed-rank test for non-normal distribution. Statistical significance was set at *p* value < 0.05.

### Ethical approval

The study protocol was approved by the local Ethics Committee (*Comitê de Ética em Pesquisa da Universidade Federal de São Paulo*, CEP-UNIFESP, CAAE: 48,643,415.2.0000.5505).

## Results

Table 1 shows the physical and biochemical parameters for the banana and control groups. Groups were comparable at baseline, except for lower concentrations of HDL-C (*p* = 0.001) and higher TG (*p* = 0.008) in the banana group. At 6-months (T6) HDL-C remained lower in banana group (*p* < 0.0001). Consumption of green-banana biomass was followed by reduction in total cholesterol (*p* = 0.010), HbA1c (*p* = 0.0007), glucose (*p* = 0.028) and non-HDL-C (*p* = 0.043), whereas we observed reduction in HbA1c (*p* = 0.002) in the diet alone group (control).

**Table 1:** Physical and biochemical parameters for banana and control groups.

variable	Control (n = 18)			Banana (n = 21)			Between groups	
	T0	T6	*p * value	T0	T6	*p *	*p* value T0	*p* value T6
TC (mg/dL)	186.8 ± 49.1	183.8 ± 35.2	0.728	185.9 ± 51.6	162.5 ± 39.0	**0.010**	0.954	0.084
HDL-C (mg/dL)	59.2 ± 13.2	57.5 ± 11.1	0.335	43.4 ± 13.8	41.1 ± 10.5	0.559	**0.001**	** < 0.0001**
LDL-C (mg/dL)	99.6 ± 34.7	83.7 ± 23.7	0.099	105.4 ± 49.0	104.9 ± 31.8	0.952	0.959	0.077
LDL-C/HDL-C ratio	1.6 (1.1–2.6)	1.8 (1.4–2.1)	0.554	2.1 (1.7–2.5)	2.0 (1.5–2.2)	0.287	0.065	0.424
non-HDL-C (mg/dL)	127.6 ± 43.3	126.3 ± 33.0	0.874	134.1 ± 44.8	113.6 ± 32.1	**0.043**	0.685	0.259
ox-LDL (mU/L)	43.6 (29.6–58.2)	38.1 (32.3—51.7)	0.138	41.6 (30.9–60.5)	42.7 (34.1–53.1)	0.651	0.977	0.544
TG (mg/dL)	97.5 (85.7–135.2)	110.0 (80.5–152.0)	0.647	160.0 (103.5–206.0)	124.0 (96.5–195.5)	0.246	**0.008**	0.118
Glucose (mg/dL)	105.5 (97.2–109.2)	97.0 (88.7–113.2)	0.269	112.0 (94.0–145.0)	103.0 (92.5–121.5)	**0.028**	0.175	0.176
HbA1c (%)	6.1 (5.9–6.7)	5.9 (5.6–6.7)	**0.002**	6.5 (6.1–7.2)	6.2 (5.8–6.6)	**0.0007**	0.110	0.264
Insulin (μUI/L)	9.0 (6.6–14.1)	10.2 (7.7–12.1)	0.990	12.7 (8.5–23.8)	14.9 (6.5–23.4)	0.658	0.127	0.094
HOMA-IR (AU)	2.3 (1.5–3.8)	2.2 (1.9–2.9)	0.861	2.4 (2.0–8.3)	4.2 (1.6–6.6)	0.287	0.105	**0.031**
Phase shift (θ)	0.005 (0.001–0.012)	0.002 (0.001–0.007)	0.203	0.006 (0.002–0.012)	0.004 (0.002–0.017)	0.719	0.707	0.286
Abs 532 nm	0.049 (0.031–0.064)	0.032 (0.021–0.064)	0.216	0.047 (0.022–0.082)	0.056 (0.027–0.088)	0.058	0.950	0.140
Abs 480 nm	0.249 (0.167–0.414)	0.203 (0.129–0.445)	0.903	0.271 (0.158–0.386)	0.292 (0.213–0.525)	**0.007**	0.983	0.349

Variables are expressed as: Mean values ± standard deviation (SD) or median [interquartile range (Q1-Q3)]. TC: total cholesterol, TG: triglycerides, non-HDL-C = sLDL-C + VLDL-C + IDL-C. Variables compared between groups using two samples *t* test or Mann–Whitney tests. Within groups comparisons were made using paired sample *t* test or Wilcoxon tests. Bold numbers: significant difference (*p* value < 0.05).

Green banana biomass was well tolerated, and no subject discontinued its use due to any side effect, such as diarrhea, constipation, or abdominal bloating. The green banana biomass was consumed at therapeutic doses and the participants were recommended to increase water intake. Both diet interventions were not only effective but closely monitored and may have reduced the impact of the active treatment.

**Figure 1:**
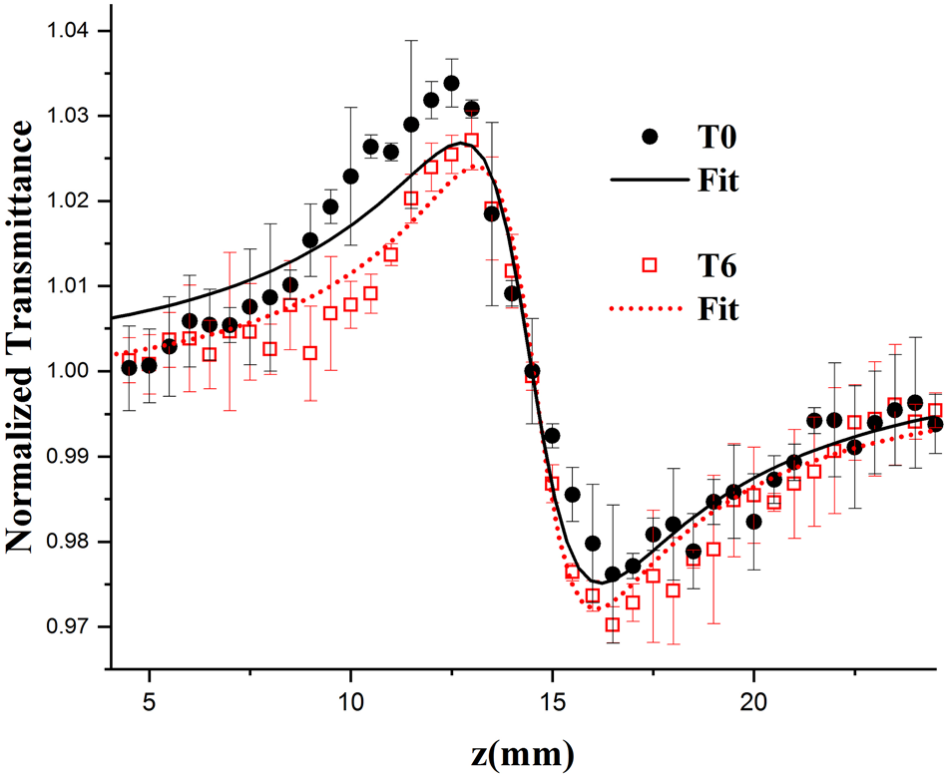
ZS typical result: Normalized transmittance as a function of the sample z position of a patient from the banana group at T0 and T6. Experimental data (symbols) and Model fits (lines).

**Figure 2:**
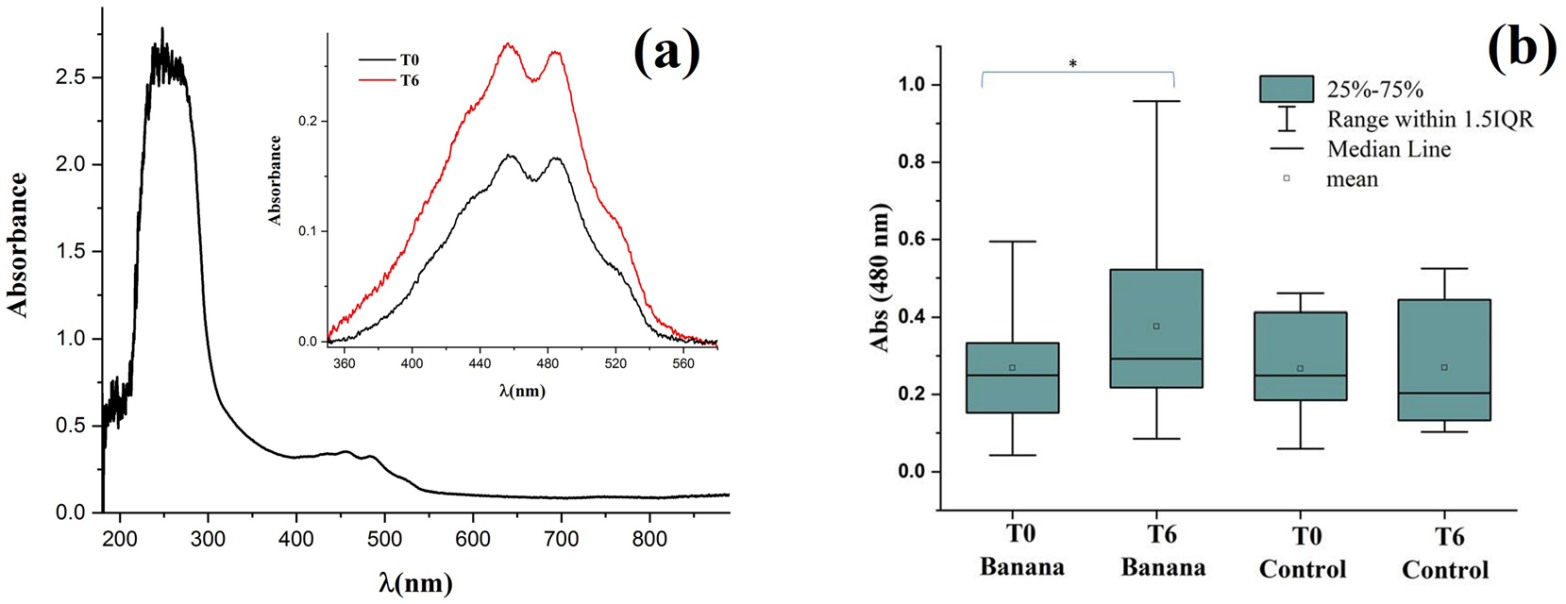
(**a**) UV–visible spectroscopy results: Typical absorbance spectra of LDL sample (patient from the banana group) at wavelengths between 200 and 900 nm. Insert: comparison with more resolution between absorbance of LDL sample from banana group at T0 and T6. (**b**) Box-plots showing the absorbance at 480 nm of the LDL of the banana and control groups at T0 and T6, *significant difference (*p* < 0.05).

Figure 1 shows the typical result of the ZS experiment of an LDL solution from a patient of the green-banana group at T0 and after T6. The results of the analysis of phase shift (θ), linear light absorbance at wavelength 532 nm (wavelength of the laser employed in the ZS experiment) and absorbance at the wavelength λ = 480 nm, characteristic of carotenoids^33,34^ are shown in Table 1. Figure 2a shows the typical extinction spectrum of the LDL sample from a patient of the banana group (wavelength range from 200 to 900 nm). In the insert of Fig. 2a we show the absorption spectra (extinction spectrum corrected by the Rayleigh scattering) of this patient in T0 and T6. Figure 2b shows the box-plot of the absorbance at wavelength 480 nm for banana and control groups at T0 and T6.

The phase shift (θ) and linear light absorption at 532 nm did not show significant difference (*p* > 0.05) between the baseline and after 6-months treatment in the banana and control groups. On the other hand, Fig. 2b shows that mean and median values of the optical absorbance at 480 nm significantly increased in the banana group after 6-months consumption of green-banana biomass (*p* = 0.007). No significant difference was observed in absorbance at 480 nm in the control group (*p* > 0.05).

**Figure 3:**
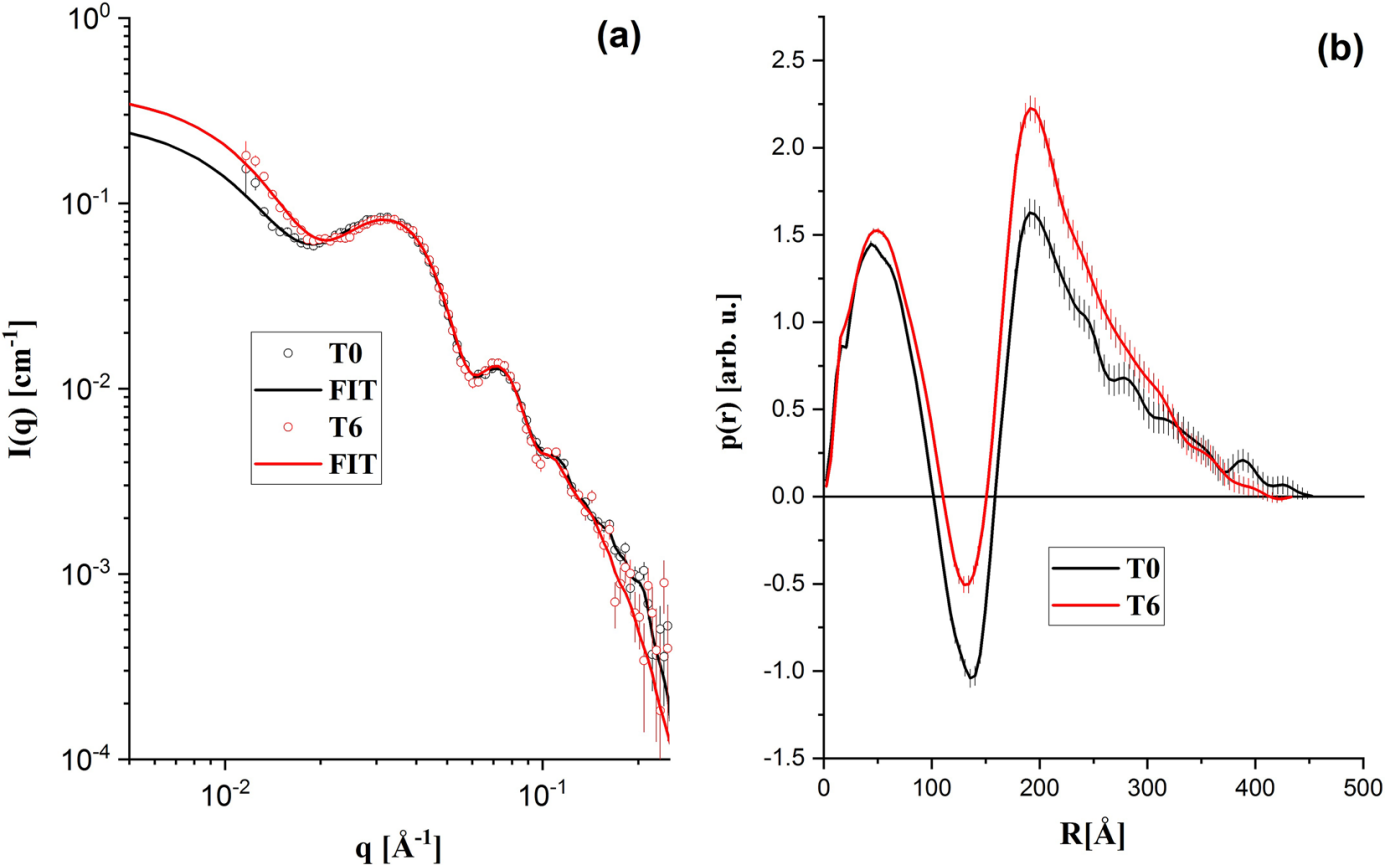
(**a**) Typical SAXS results: experimental data (open symbols) and IFT (Indirect Fourier Transformation fit—solid lines). (**b**) Calculated p(r) functions using IFT procedure. Patient from the control group at T0 and T6.

**Figure 4:**
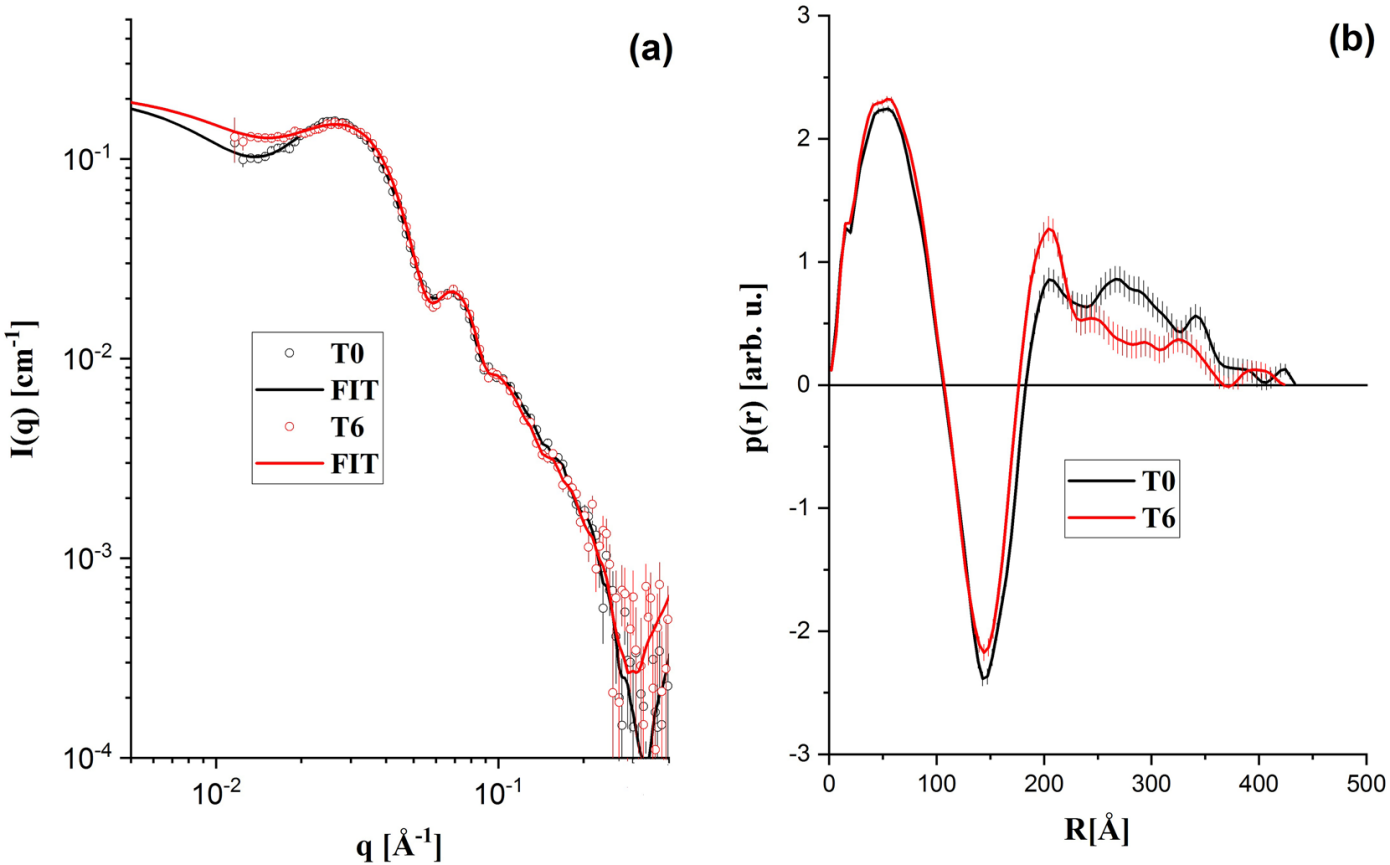
(**a**) Typical SAXS results: experimental data (open symbols) and IFT (Indirect Fourier Transformation) fit—solid lines). (**b**) Calculated p(r) functions using IFT procedure. Patient from the banana group at T0 and T6.

Figures 3 and 4 show typical SAXS results of LDL patients from the control and banana groups, respectively. No clear evidence of changes in the nanoscale of the LDL particles induced by the 6-months consumption of green-banana biomass was observed. It indicates that, at nanoscale, no detectable modifications in the mean size and shape of the LDL particles occur due to the green-banana diet. The typical diameter of the scatters in both groups obtained from the SAXS data is about 40 nm, indicating a small-scale aggregation of LDL particles (dimers, since the typical diameter of the spherical LDL is 19–25 nm)^43^.

**Figure 5:**
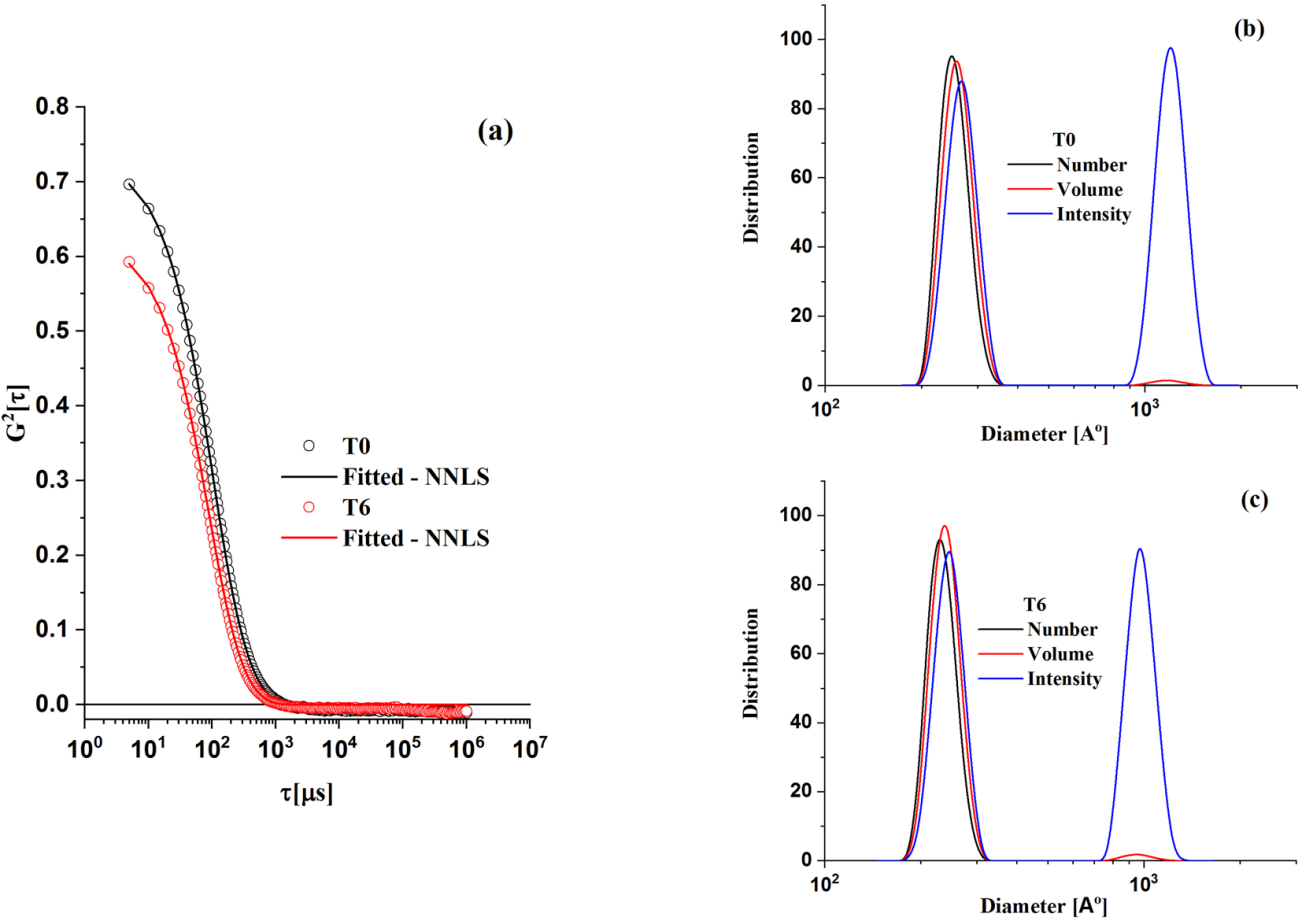
Typical DLS results: (**a**) Normalized-intensity time autocorrelation function of a patient from the banana group at T0 and T6. Solid lines are non-negative least square fits (NNLS). Size-distribution functions calculated with the data, averaged by number—black; volume—red and intensity—blue (**b**) at T0 and (**c**) T6.

The results of DLS experiments (Fig. 5) show that the average size of LDL particles in both groups, before and after the intervention in the banana group, is about 25 nm. The big peak (blue line) shown in Fig. 5b indicates that there is a small number of big aggregates in the LDL samples. Despite their small numbers, they are very efficient to scatter light. No clear differences are seen in the particle sizes and size distribution at baseline and after 6-months with green-banana biomass diet. These larger aggregates are not observed in the SAXS experiments due to the q accessible range in our setup. Values of q < 10^–2^ Å^−1^ (10^–3^ nm^−1^) would be necessary to detect them.

The results obtained in the Lipoprint setup are shown in Table 2. LDL-2 (%) subclass in control groups increased after 6-months of treatment (*p* = 0.045), however, when large LDL (%) (LDL-1 + LDL-2) was analyzed, there were not significant changes during follow-up time (*p* = 0.055). In addition, small LDL (%) (LDL-3 to LDL-7) in control group was significantly higher in comparison to the baseline time (*p* = 0.044). Despite that, both groups had similar profile after 6-months of treatment. Previously, we had demonstrated that smaller LDL was associated with increased electronegative LDL in patients with different cardiovascular risk^44^. More recently, Bhanpuri et al.^46^ investigated the effect of ketogenic diet on lipoprotein subclasses in DM2. The authors observed a modest improvement in LDL (+ 1.1%) and significant reduction in small LDL (− 20.8%) after 1 year of treatment. Although our results did not show increase in large LDL, intervention with RS was able to maintain LDL size, while hypocaloric diet treatment was associated to increased small LDL. Similar to SAXS and DLS results, the LDL size analyzed by Lipoprint system did not show differences between groups during follow-up time. Xhao et al.^45^ described that DM2 patients have smaller LDL size than non-DM2 individuals. Regarding that in our study all groups had DM2, however, in their study, the control patients were non-diabetic.

**Table 2:** Lipoprint analysis results of LDL from patients of the banana and control groups.

Variable	Control (n = 18)			Banana (n = 21)			Between groups	
	T0	T6	*p* value	T0	T6	*p* value	*p * value T0	*p* value T6
LDL-1 (%)	12.15 ± 3.04	13.01 ± 4.60	0.316	12.11 ± 3.15	12.35 ± 3.41	0.688	0.967	0.611
LDL-2 (%)	6.97 ± 2.45	8.22 ± 3.04	**0.045**	8.42 ± 2.57	9.24 ± 2.83	0.156	0.081	0.282
LDL-3 (%)	1.50 (0.82–2.42)	2.50 (0.97–3.50)	0.079	1.80 (1.25–4.35)	2.40 (1.65–5.35)	0.140	0.396	0.309
LDL-4 (%)	0.00 (0.00–0.00)	0.00 (0.00–1.10)	0.528	0.00 (0.00–0.00)	0.00 (0.00–1.05)	0.437	0.432	0.868
LDL-5 (%)	0.00 (0.00–0.00)	0.00 (0.00–0.00)	1.000	0.00 (0.00–0.00)	0.00 (0.00–0.00)	1.000	1.000	1.000
LDL-6 (%)								
LDL-7 (%)								
Large LDL (%)	19.13 ± 3.77	21.22 ± 6.29	0.055	20.53 ± 4.04	21.59 ± 3.78	0.073	0.272	0.823
Small LDL (%)	1.50 (0.82–2.42)	2.50 (1.27–4.52)	**0.044**	1.80 (1.25–4.35)	2.40 (1.65–6.95)	0.225	0.358	0.421
LDL-1 (mg/dL)	23.28 ± 10.17	24.38 ± 11.42	0.590	21.59 ± 5.79	20.12 ± 7.84	0.335	0.518	0.177
LDL-2 (mg/dL)	13.58 ± 7.11	15.43 ± 7.78	0.219	15.60 ± 6.10	14.71 ± 4.80	0.412	0.347	0.725
LDL-3 (mg/dL)	2.79 (1.65–5.37)	4.78 (1.11–6.11)	0.284	2.97 (1.85–9.01)	3.50 (2.45–7.47)	0.891	0.562	0.621
LDL-4 (mg/dL)	0.00 (0.00–0.00)	0.00 (0.00–1.72)	0.843	0.00 (0.00–0.00)	0.00 (0.00–1.30)	0.625	1.000	0.868
LDL-5 (mg/dL)	0.00 (0.00–0.00)	0.00 (0.00–0.00)	1.000	0.00 (0.00–0.00)	0.00 (0.00–0.00)	1.000	1.000	1.000
LDL-6 (mg/dL)								
LDL-7 (mg/dL)								
Large LDL (mg/dL)	36.87 ± 15.61	39.82 ± 17.51	0.338	37.19 ± 9.45	34.83 ± 9.58	0.239	0.938	0.268
Small LDL (mg/dL)	2.78 (1.64–5.37)	4.78 (2.09–7.69)	0.118	2.97 (1.85–9.49)	3.50 (2.45–8.77)	0.807	0.544	0.821
LDL size (nm)	26.9 (26.8–27.0)	26.8 (26.4–27.0)	0.105	26.8 (26.5–27.0)	26.8 (26.2–27.0)	0.832	0.267	0.571

Variables are expressed as: Mean values ± standard deviation (SD) or median [interquartile range (Q1-Q3)]. Variables compared between groups using two samples *t* test or Mann–Whitney tests. Within groups comparisons were made using paired sample *t* test or Wilcoxon tests. Bold numbers: significant difference (*p* value < 0.05).

## Discussion

Previous studies reported the effects of RS on glucose metabolism, anthropometric parameters and serum cholesterol levels in animal models, healthy or diabetic humans^4,9,47,48^. Some controversies in the results may be due to differences in the pathologic state of the subjects, source, dose and type of RS, diet composition, total dietary fiber intake and dietary RS content^49^. We did not perform a dose–response test for the green banana biomass. The dose of green banana biomass chosen in our study is in agreement with the doses reported in a recent review and meta-analysis by Falcomer^8^. The authors reported that for green banana pulp or biomass, the consumption ranged from 1.3 to 30 g/day. Our group used 40 g, equivalent to 2 tablespoons of green banana biomass, providing ~ 4.5 g of resistant starch. The effects of RS on glycemic response can be partly explained by its low rate of digestion in the small intestine in comparison with readily digestible starch^48^. Peterson et al.^50^ showed that twelve week of supplementation with RS reduced the inflammatory marker TNF-α and heart rate, but it did not significantly improve glycemic control and other CVD risk factors, in adults with pre-diabetes^50^. Different studies about the effect of RS supplementation on total cholesterol levels were done^51^. They showed that RS supplementation improves the blood-lipid profile and controls the blood-glucose levels in healthy overweight subjects, without bowel discomfort^51^.

Overall studies showed that RS intake did not affect HDL-C and TG compared with the control groups and LDL did not reveal significant heterogeneity among the trials^47^. In our study, baseline HDL-C was lower in green banana biomass group compared with controls, and remained lower after the intervention. However, in both banana biomass group and controls comparison of HDL-C values between T0 and T6 did not show differences (Table 1). Although LDL-C levels did not differ between groups and treatment arms, the non-HDL cholesterol was reduced by the treatment with green banana biomass, thus suggesting that the content of cholesterol in the atherogenic lipoproteins has decreased after green banana biomass consumption. The LDL-C/HDL-C ratio, another atherogenic risk marker, was unchanged by the treatments (Table 1).

Most of the studies about green-banana benefits for health were related to its gastrointestinal effects, followed by the glycemic/insulin metabolism, weight reduction, and renal and liver complications associated to diabetes^8^.

Our previous study showed that in individuals with diabetes the consumption of RS from green-banana biomass added to a diet for 6-months had effect on reduction in body weight, diastolic blood pressure, body mass index, waist and hip circumferences, decrease in glucose and HbA1c level, decrease in fat mass, and increase in lean mass percentage^9^. It has been proposed that the cholesterol-lowering effect observed in animal models could be attributed to the increased production of short-chain fatty acids in the large intestine^52^.

In addition to the traditional cardiovascular risk factors, the presence of small LDL particles is highly correlated to cardiovascular disease risk, in part due to their increased rate of oxidation and accessibility into the arterial sub-endothelial space for lesion development^53^. Our present results from the Lipoprint measurements showed that there is no significant difference in the percentage of large subclasses of LDL during 6-months consumption of green-banana biomass. However, control group had increased small LDL during follow-up time.

The LDL size analysis in association to SAXS, DLS and Lipoprint methods used to verify eventual nanoscale modifications of the LDL particles with the green-banana consumption did not reveal any modification of them due to the diet. By looking at all datasets, from the point of view of structural changes in the LDL particles structure, the 6-months consumption of green-banana biomass did not demonstrate any significant effect.

The parameter θ, that informs about the quality (i.e., functionality) of the LDL particles with respect to their modification, did not reveal an improvement of the LDL quality due to the green-banana diet. This result may be understood since the amplitudes of the thermal lens (represented by θ) in the banana group at T0 and T6 are very small, of the order of 10^–2^, showing no significant variation.

On the other hand, as shown in Table 1, the increase of the linear absorption at 480 nm (characteristic of carotenoids) in the banana group after the intervention was of about 7% [Abs_480_(T0) = 0.27 and Abs_480_(T6) = 0.29]. Both numbers are small (despite the fact that the difference is significant, *p* = 0.007), indicating a small number of carotenoids before and after intervention. Assuming only the β-carotene, this absorbance corresponds to about 0.86 μg/mL of this anti-oxidant in the samples investigated. In this framework, our results seem to indicate that the banana diet improved the antioxidant characteristics of the LDL of the patients upon intervention. The presence of carotenoids in the LDL is one of the responsible factors for the light absorption in the ZS experiment and, consequently, the formation of the thermal lens. The small amount of these molecules in the LDL samples is below the amount necessary to the ZS technique detects this small variation in the banana group upon intervention.

The identification of a particular molecule in a sample may be done by its light-absorption spectrum. In the case of carotenoids, which have long conjugated double bond, the main absorption bands are in the visible part of the spectrum (in some cases in the UV region). These characteristics of the spectrum inform about the chromophore of the molecule^54^.

Let us discuss now the significant increase of the absorbance at 480 nm measured in the banana group after 6-months green-banana diet. It is known that green-banana has phenolic compounds that are a natural source of antioxidants^8^. The presence of phenolic compounds was not observed in our absorbance spectra (Fig. 2a—the phenol absorbance maxima are at about 425 and 560 nm^55^, indicating that they are not incorporated in the LDL particles. This conclusion is supported by the fact that the shape of the absorbance spectra of all the patients (from both groups) observed in our study is the same. The changes are only the relative intensity of the absorbance peaks. No additional peaks or “shoulders” were observed in the spectra of patients from the banana group. Consumption of green-banana in a long time period, in our case 6-months, can increase the amount of antioxidant in the plasma. As these additional antioxidants are available in the plasma to neutralize the reactive oxygen species (ROS), the carotenoids present in the LDL may be spared, increasing their amount per LDL particle when compared to this level in patients before the treatment and that from individuals of the control group. As the LDL particles show a higher level of carotenoids in the banana group after the treatment (evidenced by the higher absorbance at 480 nm) they are more protected against oxidation, which improves their functionality (i.e., their quality—being less atherogenic).

The metabolic effects of green banana biomass cannot be explained by reduction in appetite. In our main study^9^, we compared consumption of food components of the diet, and verified that there were no differences in total energy, carbohydrate and protein intake between T0 and T6, thus ruling out the hypothesis that reduction in appetite, and less food consumption would explain our metabolic results. The composite of saciety, promoted by delayed gastric emptying, improvement in insulin resistance, as per the reduction in HbA1c and fasting glucose, reduction in non-HDL cholesterol, body weight, body mass index, fat mass and increase in lean mass, could explain the metabolic effects of green banana biomass consumption in diabetes mellitus. In addition, the increase in the absorbance at 480 nm, due to the content of carotenoids in LDL particle, can support the hypothesis of increased anti-oxidant capacity of LDL particles in subjects receiving green banana biomass.

## Conclusions

In summary, green-banana biomass, source of RS2 and natural antioxidants, clearly improves metabolic control and body composition in individuals with diabetes and pre-diabetes. In patients subjected to 6-months diet with green-banana biomass, our results show that there was an increase of the linear optical absorption of their LDL at 480 nm. This result is interpreted as an indication of the increase in the content of antioxidants (mainly carotenoids) in the LDL particles of these treated patients. LDL particles with more carotenoids are better protected against oxidative stress, being functional (i.e., less atherogenic, of better quality). These results agree with the lipoprotein subclasses observed, that remains similar to baseline time, while control group had improvement in small LDL. So, the green-banana consumption, at least in the framework of this study—during a period of 6-months, besides all the metabolic benefits already known, was shown to improve the protection of the LDL against physical–chemical modifications (oxidation and subclasses distribution), since the number of carotenoids in the particle increased. This higher protection against modifications is expected to decrease the risk of the DM2 individuals develop CVD. These benefits of the green-banana biomass encourage the use of bioactive starches with potential clinical applications in individuals with pre-diabetes and diabetes.

## Supplementary information

Supplementary file 1

## References

[1] Penn-Marshall M, Holtzman GI, Barbeau WE (2010). African Americans may have to consume more than 12 grams a day of resistant starch to lower their risk for type 2 diabetes. J. Med. Food.

[2] Ble-Castillo JL (2010). Effects of native banana starch supplementation on body weight and insulin sensitivity in obese type 2 diabetics. Int. J. Environ. Res. Public Health.

[3] Jiménez-Domínguez G (2015). Effects of acute ingestion of native banana starch on glycemic response evaluated by continuous glucose monitoring in obese and lean subjects. Int. J. Environ. Res. Public Health.

[4] Dodevska MS (2016). Effects of total fibre or resistant starch-rich diets within lifestyle intervention in obese prediabetic adults. Eur. J. Nutr..

[5] Nugent AP (2005). Health properties of resistant starch. Nutr. Bull..

[6] Jiang H (2015). Digestibility and changes to structural characteristics of green banana starch during in vitro digestion. Food Hydrocolloids.

[7] Kwak JH (2012). Dietary treatment with rice containing resistant starch improves markers of endothelial function with reduction of postprandial blood glucose and oxidative stress in patients with prediabetes or newly diagnosed type 2 diabetes. Atherosclerosis.

[8] Falcomer AL, Riquette RFR, de Lima BR, Ginani VC, Zandonadi RP (2019). Health benefits of green banana consumption: A systematic review. Nutrients.

[9] Costa, E.S., *et al.* Beneficial effects of green banana biomass consumption in patients with pre-diabetes and type 2 diabetes: A randomized controlled trial. *Br. J. Nutr.* **121**, 1365–1375 (2019). 10.1017/S0007114519000576 30887937

[10] Jin, P. & Cong, S. LOX-1 and atherosclerotic-related diseases. *Clin. Chim. Acta* **491**, 24–29 (2019). 10.1016/j.cca.2019.01.006 30639239

[11] Monteiro AM (2012). Measurement of the nonlinear optical response of low-density lipoprotein solutions from patients with periodontitis before and after periodontal treatment: Evaluation of cardiovascular risk markers. J. Biomed. Opt..

[12] Gómez S (2004). Characterization of native and oxidized human low-density lipoproteins by the Z-scan technique. Chem. Phys. Lipid..

[13] Lamarche B, Lemieux I, Despres J (1999). The small, dense LDL phenotype and the risk of coronary heart disease: Epidemiology, patho-physiology and therapeutic aspects. Diabetes Metab..

[14] Hallman DM, Brown SA, Ballantyne CM, Sharrett AR, Boerwinkle E (2004). Relationship between low-density lipoprotein subclasses and asymptomatic atherosclerosis in subjects from the Atherosclerosis Risk in Communities (ARIC) Study. Biomarkers.

[15] de Queiroz Mello AP, Albattarni G, Espinosa DHG, Reis D, Neto AMF (2018). Structural and nonlinear optical characteristics of in vitro glycation of human low-density lipoprotein, as a function of time. Braz. J. Phys..

[16] de Fatima Pedroso J (2019). Influence of Periodontal Disease on cardiovascular markers in Diabetes Mellitus patients. Sci. Rep..

[17] Martins, C. & Cardoso, S.P. Terapia nutricional enteral e parenteral: Manual de rotina técnica. In *Terapia nutricional enteral e parenteral: manual de rotina técnica* (2000).

[18] Institute of Medicine (IOM). Dietary references intakes for energy, carbohydrate, fiber, fat, fatty acids, cholesterol, protein and aminoacids (macronutrients). https://www.nap.edu/read/10490/chapter/ 1. Accessed July 31, 2019.

[19] United States Department of Agriculture: Center for Nutrition Policy and Promotion. MyPlate background. https://www.cnpp.usda.gov/Publications/MyPlate/Backgrounder.pdf. Accessed July 31, 2019.

[20] Goñi I, Garcia-Diz L, Mañas E, Saura-Calixto F (1996). Analysis of resistant starch: A method for foods and food products. Food Chem..

[21] Philippi, S. ela de Composição de Alimentos: suporte para decisão nutricional (Editora Gráfica Coronário Brasília, 2002).

[22] Instituto Brasileiro de Geografia e Estatística-IBGE. Pesquisa de Orçamentos Familiares—POF. https://www.ibge.gov.br/estatisticas/sociais/saude/24786-pesquisa-de-orcamentos-familiares-2.html?=&t=sobre. Acessed July 31, 2019.

[23] Slater B, Philippi ST, Marchioni DM, Fisberg RM (2003). Validação de Questionários de Freqüência Alimentar-QFA: Considerações metodológicas. Rev. Bras. Epidemiol..

[24] Friedewald WT, Levy RI, Fredrickson DS (1972). Estimation of the concentration of low-density lipoprotein cholesterol in plasma, without use of the preparative ultracentrifuge. Clin. Chem..

[25] Matthews D (1985). Homeostasis model assessment: Insulin resistance and β-cell function from fasting plasma glucose and insulin concentrations in man. Diabetologia.

[26] Smith PK (1985). Measurement of protein using bicinchoninic acid. Anal. Biochem..

[27] Havel RJ, Eder HA, Bragdon JH (1955). The distribution and chemical composition of ultracentrifugally separated lipoproteins in human serum. J. Clin. Investig..

[28] Sheik-Bahae M, Said AA, Wei T-H, Hagan DJ, Van Stryland EW (1990). Sensitive measurement of optical nonlinearities using a single beam. IEEE J. Quantum Electron..

[29] Santos P, Genaro-Mattos TC, Monteiro AM, Miyamoto S, Neto AMF (2012). Behavior of the thermal diffusivity of native and oxidized human low-density lipoprotein solutions studied by the Z-scan technique. J. Biomed. Opt..

[30] Layne, E. [73] Spectrophotometric and turbidimetric methods for measuring proteins. *Methods Enzymol.* **3**, 447–454 (1957).

[31] Scopes R (1974). Measurement of protein by spectrophotometry at 205 nm. Anal. Biochem..

[32] Rodriguez IR (2004). Rapid analysis of oxysterols by HPLC and UV spectroscopy. Biotechniques.

[33] Takano, H., *et al.* Involvement of CarA/LitR and CRP/FNR family transcriptional regulators in light-induced carotenoid production in Thermus thermophilus. *J. Bacteriol.* **193**, 2451–2459 (2011). 10.1128/JB.01125-10 PMC3133161 21421762

[34] Tátraaljai D, Major L, Földes E, Pukánszky B (2014). Study of the effect of natural antioxidants in polyethylene: Performance of β-carotene. Polym. Degrad. Stab..

[35] Oliveira, C.L.P. Investigating macromolecular complexes in solution by small angle X-ray scattering. Issn *Current Trends in X-ray Crystallography* (IntechOpen, 2011).

[36] Xu S, Lin B (2001). The mechanism of oxidation-induced low-density lipoprotein aggregation: An analogy to colloidal aggregation and beyond?. Biophys. J ..

[37] Berne BJ, Pecora R (2000). Dynamic Light Scattering: With Applications to Chemistry, Biology, and Physics.

[38] Pusey PN, Van Megen W (1989). Dynamic light scattering by non-ergodic media. Physica A.

[39] Zemb T, Lindner P (2002). Neutrons, X-rays and Light: Scattering Methods Applied to Soft Condensed Matter.

[40] Bro R, De Jong S (1997). A fast non-negativity-constrained least squares algorithm. J. Chemom. Soc..

[41] Hoefner DM (2001). Development of a rapid, quantitative method for LDL subfractionation with use of the Quantimetrix Lipoprint LDL System. Clin. Chem..

[42] Detection, N.C.E.P.E.P.o. & Adults, T.o.H.B.C.i. *Third report of the National Cholesterol Education Program (NCEP) Expert Panel on detection, evaluation, and treatment of high blood cholesterol in adults (Adult Treatment Panel III)*, (International Medical Pub, 2002). 12485966

[43] Esterbauer H, Gebicki J, Puhl H, Jürgens G (1992). The role of lipid peroxidation and antioxidants in oxidative modification of LDL. Free Radical Biol. Med..

[44] de Queiroz Mello AP (2010). Electronegative low-density lipoprotein is associated with dense low-density lipoprotein in subjects with different levels of cardiovascular risk. Lipids.

[45] Zhao X (2017). Analysis of lipoprotein subfractions in 920 patients with and without type 2 diabetes. Heart Lung Circ..

[46] Bhanpuri NH (2018). Cardiovascular disease risk factor responses to a type 2 diabetes care model including nutritional ketosis induced by sustained carbohydrate restriction at 1 year: An open label, non-randomized, controlled study. Cardiovasc. Diabetol..

[47] Yuan H (2018). Meta-analysis indicates that resistant starch lowers serum total cholesterol and low-density cholesterol. Nutrition research.

[48] Ble-Castillo J (2017). Acute consumption of resistant starch reduces food intake but has no effect on appetite ratings in healthy subjects. Nutrients.

[49] Gargari BP (2015). Is there any place for resistant starch, as alimentary prebiotic, for patients with type 2 diabetes?. Complement. Ther. Med..

[50] Peterson CM (2018). Effect of 12 wk of resistant starch supplementation on cardiometabolic risk factors in adults with prediabetes: A randomized controlled trial. Am. J. Clin. Nutr..

[51] Jyoshna E, Hymavathi T (2017). Study on resistant starch supplementation effect on total cholesterol: Review of literature. J. Pharmacogn Phytochem..

[52] Park OJ, Ekang N, Chang MJ, Kim WK (2004). Resistant starch supplementation influences blood lipid concentrations and glucose control in overweight subjects. J. Nutr. Sci. Vitaminol..

[53] Parish S (2012). Lipids and lipoproteins and risk of different vascular events in the MRC/BHF Heart Protection Study. Circulation.

[54] Britton G, Liaaen-Jensen S, Pfander H (2012). Carotenoids: Handbook.

[55] Pines E (2003). UV-Visible Spectra and Photoacidity of Phenols, Naphthols and Pyrenols.

