# Supplementary material for: Green-banana biomass consumption by diabetic patients improves plasma low-density lipoprotein particle functionality
Source: Sci Rep. 2020 Jul 23;10:12269. doi: 10.1038/s41598-020-69288-1 (PMC7378544; doi:10.1038/s41598-020-69288-1)
Supplement: Supplementary file 1 [file 41598_2020_69288_MOESM1_ESM.docx]

**Supplementary Material - Study Protocol**

Title

Green-banana biomass consumption by diabetic patients improves plasma low-density lipoprotein particle functionality

Authors: Zahra Lotfollahi, Ana Paula Queiroz Mello, Edna S. Costa, Cristiano L. P. Oliveira, Nagila R. T. Damasceno, Maria Cristina Izar, Antonio Martins Figueiredo Neto

**PROJECT SUMMARY**

**Objective:** The aim of this study is to investigate the effects of 6-month consumption of resistant starch (RS) from green banana biomass on the LDL particle functionality in subjects with type 2 diabetes.

**Materials and Methods:** Thirty nine (n=39) subjects, aging 60-72 years, mean age 65 years old of both sexes with diabetes (HbA1c ≥ 6.5 %) will receive nutritional support plus green-banana biomass (40 g) (n=21, Banana group) or diet alone (n=18, Control group) for 6 months. Fasting blood samples will be obtained from all participants at baseline and after 6-months. The biochemical analysis for blood including glucose, total cholesterol (TC), glycated hemoglobin (HbA1c, %), HDL-c, triglycerides (TG), LDL-c, Ox-LDL and HOMA-IR will be determined. Non-linear optical response of LDL solutions from these participants will be studied by Z-scan (ZS) technique. UV-visible spectrophotometer will be used to measure the absorbance of the LDL samples. Small Angle X-ray Scattering (SAXS) and Dynamic Light Scattering (DLS) experiments will be used to assess structural changes in LDL samples and to determine their size distribution. The Lipoprint test will be employed to determine the LDL sub-fractions in terms of sizes.

**Expected outcomes**

We expect that consumption of green-banana biomass, will effect on atherogenic lipoproteins (LDL-cholesterol, non-HDL-cholesterol), glucose metabolism (glucose, HbA1c). Due to the sensitivity of the Z-scan technique, using standard techniques such as SAXS, DLS and Lipoprint meaurements we expect to detect any changes in the LDL characteristics, specially to identify its degree of atherogenity. These results will encourage the use of bioactive starches with potential clinical applications in individuals with pre-diabetes and diabetes.

**GENERAL INFORMATION Title:** Does the green-banana biomass consumption improve the functionality of the Low-Density Lipoprotein in plasma of diabetic patients?

**Protocol identifying number**: The study protocol was approved by the local Ethics Committee (Comitê de Ética em Pesquisa da Universidade Federal de São Paulo, CEP-UNIFESP, **CAAE: 48643415.2.0000.5505**). The trial was registered (**NCT03230123**) under the acronym BIOMEL Study (Effects of Green-Banana BIOmass Consumption in Patients with Pre-diabetes and Diabetes Mellitus) acronym and can be accessed through the internet (https://clinicaltrials.gov/).

**Date:** Feberury 3^nd^ 2020

**Name and address of the sponsor / funder:**

The work was supported by the National Council for Scientific and Technological Development from Brazil, National Institute of Science and Technology Complex Fluids (INCT-FCx: 2014/50983-3 ) - (NAP-FCx: 2011.1.9358.1.6) and São Paulo Research Foundation (FAPESP –2018/07340-5 & 2016/24531-3).

INCT: Prof. Lineu Prestes Ave 580 - Zip code: 05508-000 São Paulo - SP

FAPESP: Pio XI Street 1500 – Zip code: 05468-901 São Paulo – SP.

**Name and title of the investigator(s) who is (are) responsible for conducting the research:**

Antonio Martins Figueiredo Neto

Complex Fluids Group, Instituto de Física, Universidade de São Paulo

Rua do Matão Street 1371

Zip code: 05508-090, Butantã, São Paulo - SP, Brazil

Phone: (55) 1130916830

E-mail: afigueiredo@if.usp.br

Fax number: 00551130916771, 00551130916832

**Address and telephone number(s) of the research site(s):**

1-University of São Paulo (USP) National Institute of Science and Technology Complex Fluids (INCT-FCX)

Rua do Matão Street 1371 – Zip code: 05508-090, Butantã, São Paulo - SP,

Responsibilities: Physical analysis through the Z-scan, SAXS and DLS, statistical analysis, text production.

2 – Universidade Federal de São Paulo

Rua Loefgren, 1350 – Zip code: 04040-001, Vila Clementino, São Paulo - SP,

Responsibilities: Recruitment and follow-up of patients, clinical and nutritional treatment, collection of clinical data, collection of blood samples, laboratory analysis, text production.

**RATIONALE & BACKGROUND INFORMATION**

Type 2 diabetes mellitus (DM2) is a group of metabolic disorders characterized by hyperglycemia and is the most common form of diabetes^1^. The incidence of DM2 is increasing worldwide due to modern lifestyle, in which poor dietary habits, decreased physical activity and genetic predisposition exert synergistic effect on glucose homeostasis^2^. Epidemiological data show that about 60–90% of DM2 patients are obese. It is well known that obesity is one of the major risk factors for DM2 and cardiovascular diseases (CVD)^2-4^. With the increasing number of obese and diabetic individuals, and their impact in cardiovascular diseases, there exists an intense request for healthy diets and alimentary supplements able to improve glucose metabolism and other related dysfunctions.

Resistant starch (RS) is a form of starch that resists digestion in the small intestine and is classified as a type of dietary fiber^5^. Unripe (green) banana belongs to a RS2 class, being well known as a food with low glycemic index, non-manufactured, attractive and inexpensive food containing different kinds of fibers, vitamins, minerals and bioactive compounds with high RS content^2,3,6-8^.

In a previous work, we have shown that consumption of RS was associated with significant reduction in HbA1c, fasting glucose, diastolic blood pressure, body weight, body mass index (BMI), waist and hip circumferences, fat mass percentage, and increase in lean mass percentage in subjects with diabetes and pre-diabetes^9^.

On the other side, it is well established that the presence of modified low-density lipoprotein (LDL) particles in the blood is one of the risk factors for developing CVD^10^. Modified LDL represents heterogeneous and complex sub fractions, where physicochemical changes can disrupt functional properties of this lipoprotein with impact in atherosclerosis process.

The nonlinear optical Z-scan (ZS) technique has been shown useful for evaluating the degree of modification of LDL particles in blood, in other words, the functionality of LDL particles^11^. By functionality we mean the characteristics of the native (i.e., non-modified, non-oxidized LDL) state of the particle. This term will be considered hereafter as the “quality” of the particle. This technique is often used in the physics of condensed matter and, more recently, was used to investigate the oxidation state of LDL particles^11,12^. The physical phenomenon present in these experiments is the formation of a thermal lens in a solution with the LDL when it is illuminated with a Gaussian laser beam. The amplitude of the thermal lens formed depends on the physicochemical state of the LDL particle, in which, the higher the modified (mainly oxidation) state of the particle, the smaller the amplitude of the thermal lens formed. In addition, other physical techniques such as UV-visible spectroscopy, small angle x-ray scattering (SAXS), dynamic light scattering (DLS) and the lipoprotein sub fractions determination have been used to identify modifications of the LDL particle and its association with health conditions and diseases^13-15^.

Recently the functionality of the LDL particles in diabetic patients with periodontal diseases was studied^16^. In patients with chronic periodontitis, after one-year of periodontal treatment, the functionality of the LDL particles was shown to be improved. This improvement was verified in the results obtained with the Z-Scan technique and the linear optical absorbance in the wavelength characteristic of the carotenoids present in the particles. Despite the interest in the study of the effects of the RS2 diet^8^, to the best of our knowledge, there is not a systematic study about the benefits of the green banana consumption in the functionality of the LDL in DM2 patients.

The goal of the present study is to evaluate the effect of a 6-month consumption of green-banana biomass in the LDL functionality in patients with type 2 diabetes mellitus. Besides the usual analysis of the patients’ biochemical parameters the LDL was extracted from their plasma and analyzed with different optical and scattering experimental techniques, described in the following.

**REFERENCES**

1. Penn-Marshall, M., Holtzman, G.I. & Barbeau, W.E. African Americans may have to consume more than 12 grams a day of resistant starch to lower their risk for type 2 diabetes. Journal of medicinal food 13, 999-1004 (2010).

2. Ble-Castillo, J.L., et al. Effects of native banana starch supplementation on body weight and insulin sensitivity in obese type 2 diabetics. International journal of environmental research and public health 7, 1953-1962 (2010).

3. Jiménez-Domínguez, G., et al. Effects of acute ingestion of native banana starch on glycemic response evaluated by continuous glucose monitoring in obese and lean subjects. International journal of environmental research and public health 12, 7491-7505 (2015).

4. Dodevska, M.S., et al. Effects of total fibre or resistant starch-rich diets within lifestyle intervention in obese prediabetic adults. European journal of nutrition 55, 127-137 (2016).

5. Nugent, A.P. Health properties of resistant starch. Nutrition Bulletin 30, 27-54 (2005).

6. Jiang, H., et al. Digestibility and changes to structural characteristics of green banana starch during in vitro digestion. Food Hydrocolloids 49, 192-199 (2015).

7. Kwak, J.H., et al. Dietary treatment with rice containing resistant starch improves markers of endothelial function with reduction of postprandial blood glucose and oxidative stress in patients with prediabetes or newly diagnosed type 2 diabetes. Atherosclerosis 224, 457-464 (2012).

8. Falcomer, A.L., Riquette, R.F.R., de Lima, B.R., Ginani, V.C. & Zandonadi, R.P. Health benefits of green banana consumption: a systematic review. Nutrients 11, 1222 (2019).

9. Costa, E.S., et al. Beneficial Effects of Green Banana Biomass Consumption in Patients with Pre-diabetes and Type 2 Diabetes: a Randomized Controlled Trial. British Journal of Nutrition, 1-38 (2019).

10. Jin, P. & Cong, S. LOX-1 and atherosclerotic-related diseases. Clinica Chimica Acta (2019).

11. Monteiro, A.M., et al. Measurement of the nonlinear optical response of low-density lipoprotein solutions from patients with periodontitis before and after periodontal treatment: evaluation of cardiovascular risk markers. Journal of biomedical optics 17, 115004 (2012).

12. Gómez, S., et al. Characterization of native and oxidized human low-density lipoproteins by the Z-scan technique. Chemistry and physics of lipids 132, 185-195 (2004).

13. Lamarche, B., Lemieux, I. & Despres, J. The small, dense LDL phenotype and the risk of coronary heart disease: epidemiology, patho-physiology and therapeutic aspects. Diabetes and metabolism 25, 199-212 (1999).

14. Hallman, D.M., Brown, S.A., Ballantyne, C.M., Sharrett, A.R. & Boerwinkle, E. Relationship between low-density lipoprotein subclasses and asymptomatic atherosclerosis in subjects from the Atherosclerosis Risk in Communities (ARIC) Study. Biomarkers 9, 190-202 (2004).

15. de Queiroz Mello, A.P., Albattarni, G., Espinosa, D.H.G., Reis, D. & Neto, A.M.F. Structural and Nonlinear Optical Characteristics of In Vitro Glycation of Human Low-Density Lipoprotein, as a Function of Time. Brazilian Journal of Physics 48, 560-570 (2018).

16. de Fatima Pedroso, J., et al. Influence of Periodontal Disease on cardiovascular markers in Diabetes Mellitus patients. Scientific reports 9, 1-9 (2019).

**STUDY GOALS AND OBJECTIVES**

In the literature, it is unclear whether the consumption of green banana biomass can induce modifications in the structure and functionality of lipoprotein particles in subjects with diabetes mellitus. Therefore, the objective of the present study is to evaluate the quality of the LDL particles from patients with type 2 diabetes, who will receive or not green-banana biomass during a six months’ follow-up. We will use the Z-scan technique to investigate the characteristic optical response of LDL solution samples, the UV-visible spectrophotometer to measure the linear light absorbance of LDL samples’ solutions, small angle x-ray scattering (SAXS), dynamic light scattering (DLS) and the lipoprotein sub fractions determination to identify modifications of the LDL particle.

**STUDY DESIGN**

This study is part of a 24-week, prospective, randomized, open-label trial, with parallel arms and blinded endpoints. All patients will receive nutritional couseling and intervention with green-banana biomass according to randomization. Participants will be consecutively included in the study after they read and sign the written informed consent form that will be obtained before any study procedure. The study will be conducted at the Department of Medicine, Universidade Federal de Sao Paulo, SP, Brazil. The recruitment process began in February 2016, and the intervention was conducted until March 2017. For this substudy, 39 subjects (60-72 years) mean age 65 years old of both sexes with diabetes (HbA1c ≥ 6.5 %) will receive nutritional support plus green banana biomass (40 g) (n=21) or diet alone (n=18) for 6 months.

**METHODOLOGY**

The study protocol was approved by the local Ethics Committee (Comitê de Ética em Pesquisa da Universidade Federal de São Paulo, CEP-UNIFESP, CAAE: 48643415.2.0000.5505). It was also registered (NCT03230123) under The BIOMEL Study (Effects of Green Banana BIOmass Consumption in Patients with Pre-diabetes and Diabetes MELlitus) acronym and can be accessed through the internet (<https://clinicaltrials.gov/)>.

We included 39 patients (21 patients in the Banana group and 18 patients in the Control group), of both sexes, aging 60-72 years, with diabetes (glycated haemoglobin ≥ 6.5%) and pre-diabetes (HbA1c between 5.7% and 6.4%), receiving a stable dose of anti-hyperglycemic drugs. Patients under insulin therapy, or those that during the study needed change in dose or addition of medication for diabetes were excluded. Neoplasms, except basal-cell carcinoma, heart (NYHA class III or IV) and renal failure (e-GFR < 30 mL/min) or dialysis therapy, AIDS, uncontrolled hypothyroidism (TSH > 10 μUI/mL), active liver disease, severe psychiatric disorders, or any other disease that, in the investigator’s opinion, could interfere with the results were also excluded. This trial was approved by local Human Ethics Committee and all patients signed the formal consent before data collection. The calculation of sample size took into account variations in HbA1c between groups, with a type I error α of 0.05 and a type II error β of 0.2 (80 % power).

The diet plan will be individualized according to the Brazilian Society of Diabetes, taking into account the total energy expenditure (TEE), with standardized menus for weight reduction (20-25 kcal/kg of current weight), weight maintenance (25-30 kcal/kg of current weight), and weight gain (30-35 kcal/kg of current weight), according to patient characteristics. Macronutrients intake will be accessed by the Acceptable Macronutrient Distribution Range. Substitutions by equivalent foods will be made using a food replacement list (FRL).

**Diet Plan**

1- Green-banana biomass

Green-banana biomass is added to any food preparation without heating as 2 tablespoons (40 g) per day, providing ~ 4.5 g of RS. Nutrition information and the amount of RS in green-banana biomass have been previously described and can be found elsewhere^9^.

2- Evaluation of food consumption

Food consumption will be estimated by 24 h food records and standardized food-frequency questionnaires (FFQs) will be obtained at baseline and 6 months, with total energy intake, macro- and micro-nutrients, lipids, cholesterol, carbohydrates, fatty acids, and vitamins calculated by the Avanutri Software (Avanutri Revolution, v. 4.0).

**Biochemical analysis**

Fasting blood samples will be obtained from all participants at baseline and after 6-months. Commercial kits (Cobas Mira, Roche, Switzerland) will be used in the analysis for blood glucose, glycated hemoglobin (HbA1c, %), total cholesterol (TC), HDL-c, and triglycerides (TG). LDL-c concentration will be calculated by the Friedewald equation. Fasting insulin will be measured using the immunofluorometric assay. The HOMA-IR (fasting insulin (μUI/mL) × fasting glucose (mg/dL)/405) will be calculated, with the cutoff value set at ≥ 2.825. Protein concentrations in LDL particle will be determined by using the bicinchoninic acid (BCA) method, Pierce BCA Protein Assay kit (Thermo Fisher Scientific, MA, USA) with bovine serum albumin (BSA) as standard.

**Low-Density Lipoprotein Separation**

Blood will be collected and immediately separated in plasma and stored at −80°C until analysis. Low-density lipoprotein will be isolated from plasma by preparative sequential ultracentrifugation (18 h, 105,000×g, 4 °C), using a density cut-off point of 1.063 g/mL, by ultracentrifuge equipped with a fixed-angle rotor (Hitachi Himac CP 70MX, Tokyo, Japan). These samples will be dialyzed against PBS with EDTA (pH 7.4, 4 °C, 12 h, with agitation) to remove the salts.

**Z-Scan technique**

The Z-scan (ZS) is an experimental technique to measure nonlinear optical properties of materials. In this technique, the LDL solution sample is encapsulated between two micro-slides glass with a spacer of 200 μm and is illuminated by a focused laser beam (wavelength 532 nm, power 100 mw), propagating in the z direction. A mechanical chopper provides light pulses of about 30 milliseconds. The sample moves along the z-axis before and after the focal point z=0 and the transmitted-laser beam is detected by a detector. The normalized transmittance as a function of the sample-z position is calculated dividing the voltage on the photodetector at each z-position of sample by the voltage when the sample is at a position far from the focal point. The typical result in the ZS experiment is a peak to valley (or valley to peak) curve. This peak to valley amplitude ($\Delta\Gamma_{pv}$) is proportional to the phase shift (θ) of the thermal lens formed.

**UV-Visible Spectroscopy**

The spectrophotometer measures the intensity of light passing through a sample and compares it to the intensity of the incident light beam. The linear-absorbance spectra are measured by a UV-visible spectrophotometer with light wavelength from 200 nm to 900 nm, using deuterium and tungsten halogen light sources and a spectrometer (USB4000, from Ocean Optics) connected to a computer for data acquisition. The samples are conditioned into a quartz cuvette, with optical path length of 1 cm. The spectrophotometer measures the extinction spectrum, which is the sum of both the Rayleigh scattering and the absorbance. The Rayleigh scattering is proportional to λ^-4^, and its intensity is estimated for each one of the samples. The absorbance is then calculated removing the scattering contribution from the extinction spectra. As is known, the LDL particle contains various molecules, and each of them has different absorption spectra. In the present study we will investigate the absorbance values of LDL solution samples at two wavelength: 1) λ=480 nm, the wavelength corresponding to the maximum of the absorbance spectrum of Carotenoids, and 2) λ=532 nm, wavelength as used for the Z-scan experiment.

**Small Angle X-ray Scattering (SAXS)**

Small-angle X-ray scattering (SAXS) is a standard technique that can be applied to the study of particles in solution, providing information about size, shape, polydispersity, flexibility, oligomerization and aggregate state. SAXS data will be collected in a Xenocs-XEUSS diffractometer. X-rays (wavelength λ = 1.54 Å Cukα) are collimated by two sets of scatter-less slits and reaches the LDL sample placed in a cylindrical borosilicate glass capillary. This capillary is mounted on a homemade stainless-steel case, which allows an easy handling, wash and rinse. Therefore, the lipoproteins and the corresponding buffers can be measured in the same conditions. The two-dimensional scattering patterns will be registered by a detector. The images will be integrated with the Fit2D software and the data treatment will be performed using standard procedures

**Dynamic Light Scattering (DLS)**

The DLS, known as photon correlation spectroscopy, is used to assess eventual aggregation of particles and the LDL size distributions. DLS measurements will be carried out using a 90Plus particle Size Analyser (Brookhaven, Holtsville, NY, USA). In this technique, the sample is illuminated by a laser beam (wavelength 657 nm and power of 35 mW) and the fluctuations of the scattered light are detected by a fast photon detector positioned at 90° from the incident light direction. The DLS measurements provide intensity correlation functions that are analyzed to determine the particle-size distribution, weighted by number, volume, and intensity of scattered light. The fits will be obtained by using the NNLS (non-negative least squares) method.

**Lipoprint system**

The lipoprotein fractions (VLDL and IDL) and sub-fractions of LDL will be determined by the Lipoprint system (Quantimetrix, Redondo Beach, CA), which is based on the separation and quantification of lipoprotein sub-fractions by non-denaturing polyacrylamide tube gel electrophoresis. To perform this procedure, 25 μL of the serum or plasma is added to the polyacrylamide gel tube and 200 μL of the dye-gel solution. The sample is homogenized. Then the tubes containing the samples are photo-polymerized and subjected to the electrophoresis process. After separation of the sub-fractions, the tubes are scanned in order to identify each sub-fraction. The LDL1 and LDL2 sub-fractions are classified as Large-LDL (phenotype A) and sub-fractions LDL3 to LDL7 are classified as smaller and denser particles (Small-LDL or phenotype non-A). The LDL phenotypes are based in cut-off points (phenotype A ≥ 26.8 nm and phenotype non-A<26.8 nm)13,14,42. All analyses will be conducted in duplicate and coefficients of variance intra and inter assay are between 1-15%.

**SAFETY CONSIDERATIONS**

All the participants will be informed about the objectives and methodology of the work in an accessible language, through the term of free and informed consent. All participants will receive nutrition counseling and medical treatment. Blood samples will be collected by a trained professional to avoid discomfort to participants.

**FOLLOW-UP**

The present study had a follow-up of 24-weeks for clinical, nutritional and laboratory parameters, as well as to physical data.

**DATA MANAGEMENT AND STATISTICAL ANALYSIS**

Numerical variables will be expressed as means ± Standard Deviation (SD) for normal distribution and median (Inter-Quartile Range (IQR=Q1-Q3)) for non-normal distribution.

Shapiro-Wilk test will be used to verify normality of the data distribution. For comparison between groups, unpaired T-test, for normal distribution or Mann-Whitney test, for non-normal distribution, will be used. Within-group comparisons will be carried out using the paired T-test, to compare groups with normal distribution, or Wilcoxon signed-rank test for non-normal distribution. Statistical significance is set at p-value < 0.05.

**QUALITY ASSURANCE**

This clinical study with a follow-up of 24-weeks was registered at clinical trials (NCT03230123) under The BIOMEL Study (Effects of Green Banana BIOmass Consumption in Patients with Pre-diabetes and Diabetes MELlitus) acronym and can be accessed through the internet (<https://clinicaltrials.gov/)>. The study protocol was approved by the local Ethics Committee (Comitê de Ética em Pesquisa da Universidade Federal de São Paulo, CEP-UNIFESP, CAAE: 48643415.2.0000.5505).

**EXPECTED OUTCOMES OF THE STUDY**

The results of the present study may interfere in the treatment of diabetic patients to improve lipid profile, lipoprotein quality, as well as prevent cardiovascular diseases. The importance of the green-banana biomass for the treatment of subjects with diabetes can be emphasized to prevent and improve lipoprotein quality parameters related to cardiovascular diseases. Moreover, public health policies may use preventive methods for systemic diseases nutrition therapies. Due to the sensitivity of the z-scan technique, we expect we will be able to verify changes in the LDL characteristics, specially to identify its degree of atherogenicity. This multidisciplinary approach broads the horizons of the conclusions of the work, emphasizing the benefits of the addition of green-banana biomass to the diet with respect to the development of CVD in diabetic patients.

**DISSEMINATION OF RESULTS AND PUBLICATION POLICY**

The results of the present study will be disseminated to scientific community through the publication of dissertations, theses, scientific papers and books. Participants will have access to the results obtained as well as the benefits that treatment with green- banana biomass can bring to cardiovascular health. Lectures and seminars can be organized to disseminate the findings to the local scientific community.

The following authors will be recognized in the publications related to this research: Zahra Lotfollahi, Ana Paula P. Q. Mello, Edna S. Costa, Cristiano L. P. Oliveira, Nagila R. T. Damasceno, Maria Cristina Izar.

Antonio Martins Figueiredo Neto, being the latter, responsible for conducting the publication.

**DURATION OF THE PROJECT**

The research had a total duration of 24-weeks, each phase of the project being divided as follows: Recruitment of patients: 8 months. Collection of blood samples: 2 weeks. Nutritional treatment: 6 months. Clinical data collection: 12 months. Laboratory tests: 6 months. Organization of data and statistical analysis: 3 months. Production of the text: 3 months.

**PROBLEMS ANTICIPATED:** nothing

**PROJECT MANAGEMENT**

Zahra Lotfollahi: literature search, experimental studies, data acquisition and data analysis, statistical analysis, manuscript preparation and manuscript review.

Ana Paula P. Q. Mello: experimental studies and data analysis.

Edna S. Costa: experimental studies and data analysis.

Cristiano L. P. Oliveira: experimental studies and data analysis, manuscript preparation, manuscript editing and manuscript review.

Nagila R. T. Damasceno: experimental studies and data analysis, manuscript preparation, manuscript editing and manuscript review.

Maria Cristina Izar: definition of intellectual content, concepts, design, manuscript preparation, manuscript editing and manuscript review.

Antonio Martins Figueiredo Neto: definition of intellectual content, concepts, design, manuscript preparation and manuscript review.

**ETHICS**

All procedures received approval from the local Ethics Committee (Comitê de Ética em Pesquisa da Universidade Federal de São Paulo, CEP-UNIFESP, CAAE: 48643415.2.0000.5505).

The participation of the research subject does not imply an increased risk to any occurrence that no longer inherent in the treatment itself such as mild or moderate local pain during blood collection. In case of other reasons of force majeure, the participation of the subject of the search will be terminated immediately.

All co-authors received the manuscript, describing the objectives, risks and benefits of the research, in non-technical language. After reading the free and informed consent term, possible doubts were considered. Co-authors who agreed to participate in the study signed the term.

**INFORMED CONSENT FORMS**

All authors declare no conflicts of interest with the presente work.
